# The Effect of Orally Administered Dronabinol on Optic Nerve Head Blood Flow in Healthy Subjects—A Randomized Clinical Trial

**DOI:** 10.1002/cpt.1797

**Published:** 2020-02-23

**Authors:** Nikolaus Hommer, Martin Kallab, Stephan Szegedi, Stefan Puchner, Kristina Stjepanek, Martin Bauer, René M. Werkmeister, Leopold Schmetterer, Marihan Abensperg‐Traun, Gerhard Garhöfer, Doreen Schmidl

**Affiliations:** ^1^ Department of Clinical Pharmacology Medical University of Vienna Vienna Austria; ^2^ Center for Medical Physics and Biomedical Engineering Medical University of Vienna Vienna Austria; ^3^ Singapore Eye Research Institute Singapore Singapore; ^4^ Lee Kong Chian School of Medicine Nanyang Technological University Singapore Singapore; ^5^ Ophthalmology and Visual Sciences Academic Clinical Program Duke‐NUS Medical School Singapore Singapore; ^6^ SERI‐NTU Advanced Ocular Engineering (STANCE) Singapore Singapore; ^7^ Institute of Ophthalmology Basel Switzerland; ^8^ Department of Child and Adolescent Psychiatry Medical University of Vienna Vienna Austria

## Abstract

It has been hypothesized that besides its intraocular pressure (IOP) lowering potential, tetrahydrocannabinol (THC) may also improve ocular hemodynamics. The aim of the present study was to investigate whether single oral administration of dronabinol, a synthetic THC, alters optic nerve head blood flow (ONHBF) and its regulation in healthy subjects. The study was carried out in a randomized, placebo‐controlled, double‐masked, two‐way crossover design in 24 healthy subjects. For each study participant, 2 study days were scheduled, on which they either received capsules containing 5 mg dronabinol or placebo. ONHBF was measured with laser Doppler flowmetry at rest and while the study participants performed isometric exercise for 6 minutes to increase mean arterial blood pressure (MAP). This was repeated 1 hour after drug intake. Ocular perfusion pressure (OPP) was calculated as 2/3MAP–IOP. Dronabinol was well tolerated and no cannabinoid‐related psychoactive effects were reported. Neither administration of dronabinol nor placebo had an effect on IOP, MAP, or OPP. In contrast, dronabinol significantly increased ONHBF at rest by 9.5 ± 8.1%, whereas placebo did not show a change in ONHBF (0.3 ± 7.4% vs. baseline, *P* < 0.001 between study days). Dronabinol did not alter the autoregulatory response of ONHBF to isometric exercise. In conclusion, the present data indicate that low‐dose dronabinol increases ONHBF in healthy subjects without affecting IOP, OPP, or inducing psychoactive side effects. In addition, dronabinol does not alter the autoregulatory response of ONHBF to an experimental increase in OPP. Further studies are needed to investigate whether this effect can also be observed in patients with glaucoma.

**Study Highlights**

**WHAT IS THE CURRENT KNOWLEDGE ON THE TOPIC?**

☑ Optic nerve head blood flow (ONHBF) is impaired in patients with glaucoma. Tetrahydrocannabinol (THC) is used in the treatment for glaucoma in some countries for several years due to its intraocular pressure (IOP) lowering effect. Beside its IOP lowering potential, THC features neuroprotective effects and may improve ocular hemodynamics.

**WHAT QUESTION DID THIS STUDY ADDRESS?**

☑ Does a cannabinoid receptor agonist (dronabinol) alter ONHBF and vascular autoregulation?

**WHAT DOES THIS STUDY ADD TO OUR KNOWLEDGE?**

☑ The present data indicate that orally administered, low‐dose dronabinol increases ONHBF in healthy subjects without affecting IOP, ocular perfusion pressure, or inducing psychoactive side effects.

**HOW MIGHT THIS CHANGE CLINICAL PHARMACOLOGY OR TRANSLATIONAL SCIENCE?**

☑ Further studies are needed to investigate whether this effect also occurs in patients with ocular vascular disease or glaucoma. If this is the case, THC may become an alternative therapy approach.

There is evidence that optic nerve head blood flow (ONHBF) is autoregulated in response to changes in ocular perfusion pressure (OPP), meaning that ONHBF remains relatively stable whereas OPP increases or decreases.^1^, ^2^, ^3^, ^4^, ^5^, ^6^, ^7^ There is general agreement now that this physiological response of the ocular vasculature to keep blood flow constant during changes in OPP is disturbed under several pathological conditions, including glaucoma.^8^ Impaired ONHBF autoregulation has been hypothesized to lead to optic nerve head (ONH) ischemia and, in turn, to axonal damage and subsequent loss of retinal ganglion cells.^9^, ^10^

Recently, the cannabinoid system has received much attention as a possible drug target for glaucoma.^11^ Cannabinoid receptors have been consistently identified in several tissues of the human eye, such as the ciliary epithelium, the trabecular meshwork, Schlemm’s canal, ciliary muscle, ciliary body vessels, and the retina. It is known that activation of cannabinoid receptors decreases aqueous humor production and increases trabecular and uveoscleral outflow, thereby reducing intraocular pressure (IOP).^12^ Recent studies suggest that activation of cannabinoid receptor may feature IOP independent effects, such as neuroprotective properties or improvement in ocular hemodynamics.^13^, ^14^, ^15^, ^16^, ^17^ In particular, the finding that activation of cannabinoid receptors show vasoactive actions in isolated perfused retinal arterioles makes it an interesting target to alter ocular blood flow independent of IOP.^18^

Thus, the primary aim of the present study was to investigate whether administration of a cannabinoid receptor agonist alters ONHBF. As a secondary outcome, the effect of a cannabinoid receptor agonist on the vascular autoregulatory response of the ONH to changes in OPP was assessed. For this purpose, we used dronabinol, a synthetic tetrahydrocannabinol derivate ((–)‐trans‐Δ^9^‐Tetrahydrocannabinol) that is legally available in most European countries and received drug approval from the US Food and Drug Administration (FDA) in 1985 and from the European Medicines Agency (EMA) in 2010 for the treatment of anorexia due to AIDS, pain, and nausea and vomiting related to cancer treatment in patients who have failed to respond adequately to conventional antiemetic treatments.^19^, ^20^ As dronabinol is a registered drug, it is known to have an excellent safety profile and allows for exact and reliable dosing. In the current study, a single 5 mg dose of dronabinol was administered to healthy subjects in a randomized, controlled, double‐masked, two‐way crossover study design and ocular as well as systemic hemodynamic parameters were measured. To assess ONH autoregulation, OPP was increased by isometric exercise and ONHBF was measured continuously to quantify the autoregulatory response before and after drug administration.

## Materials and Methods

### Subjects

The present study was conducted in compliance with the Declaration of Helsinki and the Good Clinical Practice guidelines of the European Union and was approved by the Ethics Committee of the Medical University of Vienna. Written informed consent was obtained from all participants. Men and women were included in equal parts.

The following examinations and tests were carried out in each subject in the 4 weeks before the first study day: Medical history, pregnancy test in women of childbearing potential, urine drug test and analysis (white blood cell count, nitrite, pH, protein, glucose, ketones, urobilinogen, bilirubin, and blood/hemoglobin), alcohol breath test, physical examination, including 12‐lead echocardiogram and measurement of systemic hemodynamics, blood draw for hematological status (hemoglobin, hematocrit, red blood cell (RBC), mean corpuscular hemoglobin, white blood cell count, platelet count, activated partial thromboplastin time, and thrombin time), clinical chemistry (sodium, potassium, creatinine, GPT (ALAT), γ‐GT, total bilirubin, and total protein), and hepatitis B and C, and HIV‐Serology, a psychiatric examination using the modified Structured Clinical Interview^21^, ^22^ test and an ophthalmic examination (best corrected visual acuity, slit lamp biomicroscopy, indirect funduscopy, and measurement of IOP).

If any clinically significant abnormality was found as part of the prestudy screening, including history of drug or alcohol abuse, the subject was not included. Only subjects with ametropia of less than six diopters were allowed to participate in the present study. Further exclusion criteria were regular use of medication, any drug intake during the 3 weeks before the start of the study (except contraceptives), and smoking.

### Study design

**Figure 1:**
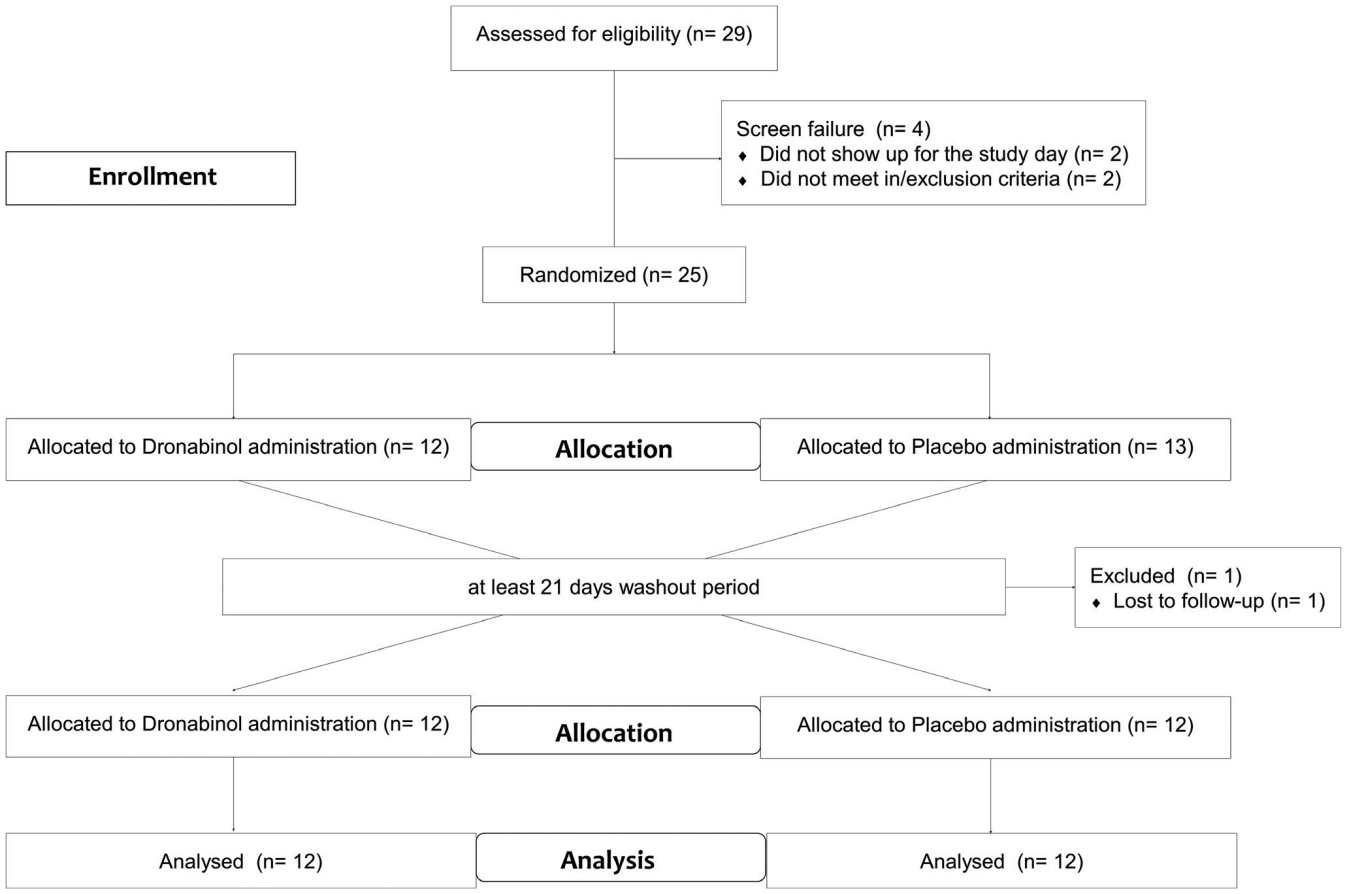
Illustration of the study design. THC, tetrahydrocannabinol.

The study was conducted in a randomized, double‐masked, placebo‐controlled, two‐way crossover design. Randomization lists were generated by a computer software (http://www.randomization.com) using the method of randomly permuted blocks by a member of the Department of Clinical Pharmacology not involved in the study procedures. According to this randomization list, a randomization envelope for each subject was prepared. Sample size calculation was based on previous measurements of ONH blood flow.^3^ Given the variability observed in our previous experiments (SD of ~ 10%), an alpha error of 0.05 and a power of 0.80, a sample size of 24 healthy subjects allowed us to detect changes in ONH blood flow of 8% or less. Subjects were randomly assigned to receive dronabinol capsules on one study day and placebo capsules identical in appearance on the other study day. A washout period of at least 21 days was scheduled between the two study days (**Figure** 1).

At the beginning of each of the two study days, a pregnancy test was performed in women of childbearing potential. A urine drug screening test as well as an alcohol breath test were performed in each subject. Afterward, one drop of tropicamide 0.5% was instilled in the randomly chosen study eye.

After a resting period of at least 20 minutes, ONHBF was measured continuously for 3 minutes at baseline. Thereafter, subjects performed isometric exercise (squatting) for 6 minutes and ONHBF was measured continuously. Systemic hemodynamics were assessed every minute during this procedure. IOP was measured before and at the end of the squatting period. Thereafter, a resting period of at least 30 minutes was scheduled.

Then, subjects received dronabinol on one study day and placebo on the other study day according to the randomization list together with a standardized meal containing of 15 g butter, 2 pieces of bread, and 250 mL of water to assure comparable bioavailability. One hour after drug administration, ONHBF measurements as described above were repeated.

### Investigational medical product and placebo

#### Dronabinol, Bionorica ethics, Neumarkt, Germany

Capsules containing 5 mg dronabinol, also referred to as (–)‐trans‐Δ^9^‐tetrahydrocannabinol; dose: one capsule (total dose 5 mg). This dose was chosen based on the recommended starting dose outlined in the product brochure. Placebo capsules were identical in appearance to dronabinol capsules without an active ingredient. Dronabinol and placebo capsules were produced and provided by Allerheiligen Apotheke, Mag. pharm. Herbert Baldia KG, Allerheiligenplatz 4, 1200 Vienna under good manufacturing practice conditions.

### Methods

#### Noninvasive measurement of systemic hemodynamics

Systolic, diastolic, and mean arterial pressure (SBP, DBP, and MAP) were measured on the upper arm by an automated oscillometric device. Pulse rate was automatically recorded from a finger pulse‐oximetric device (Infinity Delta, Draeger, Luebeck, Germany).

#### Intraocular pressure and ocular perfusion pressure

IOP was measured using a slit‐lamp mounted Goldmann applanation tonometer. Before each measurement, one drop of oxybuprocaine hydrochloride combined with sodium fluorescein was used for local anesthesia of the cornea. OPP was estimated as 2/3 MAP‐IOP.^23^

#### Laser Doppler flowmetry

ONHBF was measured by laser Doppler flowmetry (LDF).^24^, ^25^ Using this method, the vascularized tissue was illuminated by coherent laser light. Scattering on moving RBCs leads to a frequency shift in the scattered light, whereas static tissue components do not change light frequency. Instead, these lead to randomization of light directions impinging on RBCs. This light diffusing in vascularized tissue induces a broadening of the spectrum of scattered light, from which mean RBC velocity, blood volume, and the blood flow can be calculated in arbitrary units. In the present study, LDF was performed at the inferior temporal neuroretinal rim to assess ONH blood flow and care was taken that approximately the same location was used for all measurements.

### Data analysis

The polynomial correction approach was used to correct for unstable reference signals (direct current).^26^ Briefly, the parameter “yield” was calculated as direct current/gain. A regression model applying a third‐order polynomial equation was used on the logarithmic values of yield and the respective LDF parameters to calculate the corrected LDF data.

An analysis of variance (ANOVA) model was applied to detect differences in the time course of OPP, IOP, MAP, heart rate (HR), SBP, and DBP between dronabinol and placebo. Within the ANOVA model, predefined planned comparisons were performed to detect differences between time points and groups. The choice of comparisons was defined in the study protocol and determined before study start.

For analysis of ONHBF, average data over 1 minute were used. A repeated‐measures ANOVA model was applied. Differences between the treatments were calculated based on the interaction between time and treatment. For this purpose, the time effect was used to characterize the effect of squatting on the outcome parameters. Again, predefined planned comparisons were performed within the model to assess differences within groups and time points.

In addition, pressure/flow relationships were calculated. For this purpose, the data were expressed as percentage of change in OPP and flow values over baseline. OPP values were then sorted according to ascending values and grouped into six groups. As such, 24 values were classified in each of the groups. A statistically significant deviation from baseline flow was defined when the 95% confidence interval did not overlap with the baseline value anymore.^27^, ^28^

For data description, percentage of changes over baseline were calculated. A *P* value < 0.05 was considered the level of significance. Statistical analysis was carried out using CSS Statistica for Windows (Statsoft, version 6.0, Tulsa, CA).

## Results

No adverse effects or cannabinoid‐related psychoactive effects of the study medication were observed or reported by the subjects, and all study procedures were well tolerated. No cannabinoid‐induced change in HR was observed (**Table** 1). None of the subjects had any clinically relevant changes in laboratory parameters, 12‐lead echocardiogram, or the physical examination at the follow‐up visit.

**Table 1:** Resting values before isometric exercise for both study days before administration of placebo or dronabinol (predose) and after drug administration (postdose).

	Placebo day (*n* = 24)	Dronabinol day (*n* = 24)	*P* value
Systolic blood pressure (mmHg)			
Predose	124 ± 8	121 ± 11	0.32
Postdose	124 ± 10	124 ± 10	0.50
*P* value	0.42	0.16	
Diastolic blood pressure (mmHg)			
Predose	80 ± 8	78 ± 8	0.28
Postdose	78 ± 8	79 ± 8	0.75
*P* value	0.24	0.63	
Mean arterial pressure (mmHg)			
Predose	97 ± 8	95 ± 10	0.42
Postdose	96 ± 8	95 ± 9	0.73
*P* value	0.76	0.66	
Heart rate (bpm)			
Predose	65 ± 12	65 ± 11	0.85
Postdose	65 ± 13	65 ± 11	0.94
*P* value	0.86	0.81	
Intraocular pressure (mmHg)			
Predose	14 ± 3	13 ± 3	0.57
Postdose	13 ± 3	13 ± 3	0.58
*P* value	0.36	0.31	
Ocular perfusion pressure (mmHg)			
Predose	52 ± 6	51 ± 6	0.46
Postdose	51 ± 5	51 ± 5	0.80
*P* value	0.14	0.64	
Optic nerve head blood flow (a.u.)			
Predose	23 ± 4	25 ± 7	0.40
Postdose	23 ± 4	27 ± 7	0.04
*P* value	0.97	< 0.001	

Data are presented as mean ± SD.

Twenty‐four healthy subjects, of which 12 were women, aged between 20 and 35 years (mean 26 ± 4 years) finished the study according to the protocol (**Figure** 1). Baseline values for both study days are given in **Table** 1. No significant differences in baseline parameters were observed on the 2 study days.

**Figure 2:**
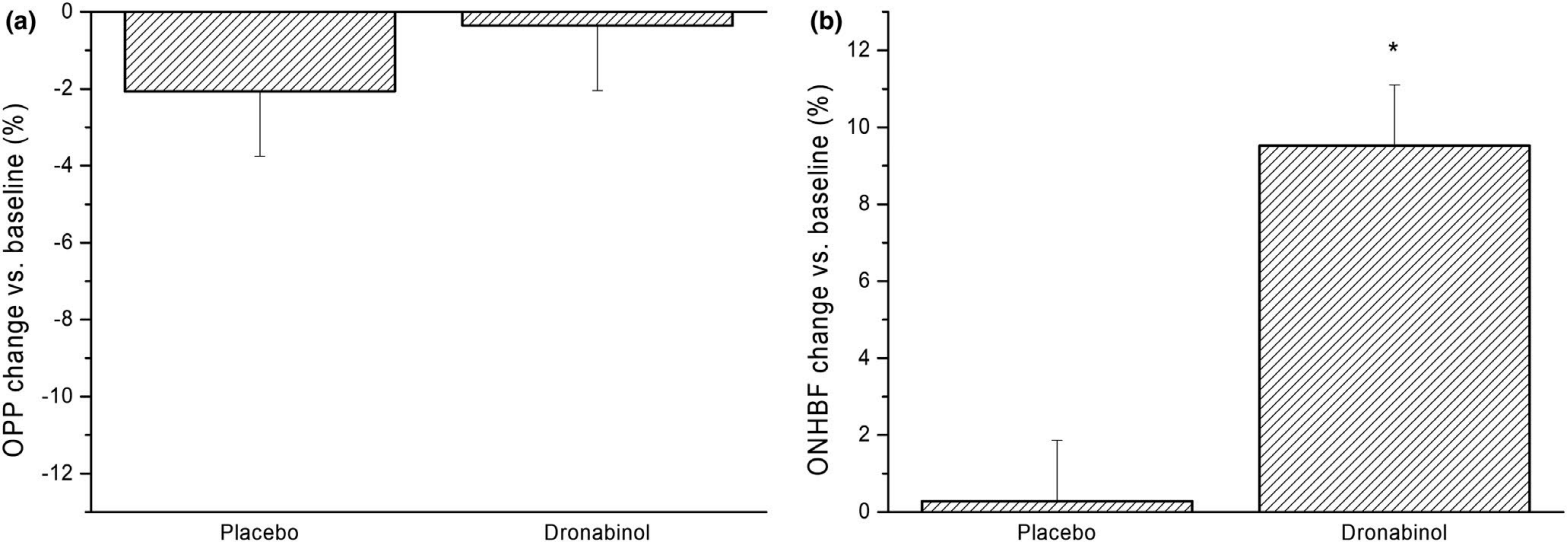
(**a**) Relative change in ocular perfusion pressure (OPP) and (**b**) optic nerve head blood flow (ONHBF) after administration of placebo and dronabinol. Data are presented as mean ± SD (*n* = 24). *Significant changes vs. baseline.

Neither administration of placebo nor of dronabinol had an effect on resting SBP, DBP, HR, or IOP (**Table** 1). No significant change in OPP was observed (−2.1 ± 8.4% after administration of placebo, −0.4 ± 8.2% after administration of dronabinol, *P* = 0.48 between study days, **Figure** 2 **a**). Although placebo had no effect on ONHBF at rest (+0.3 ± 7.4% vs. baseline), intake of dronabinol induced a significant increase in ONHBF (+9.5 ± 8.1% vs. baseline, *P* < 0.001 between groups, **Figure** 2 **b**).

**Figure 3:**
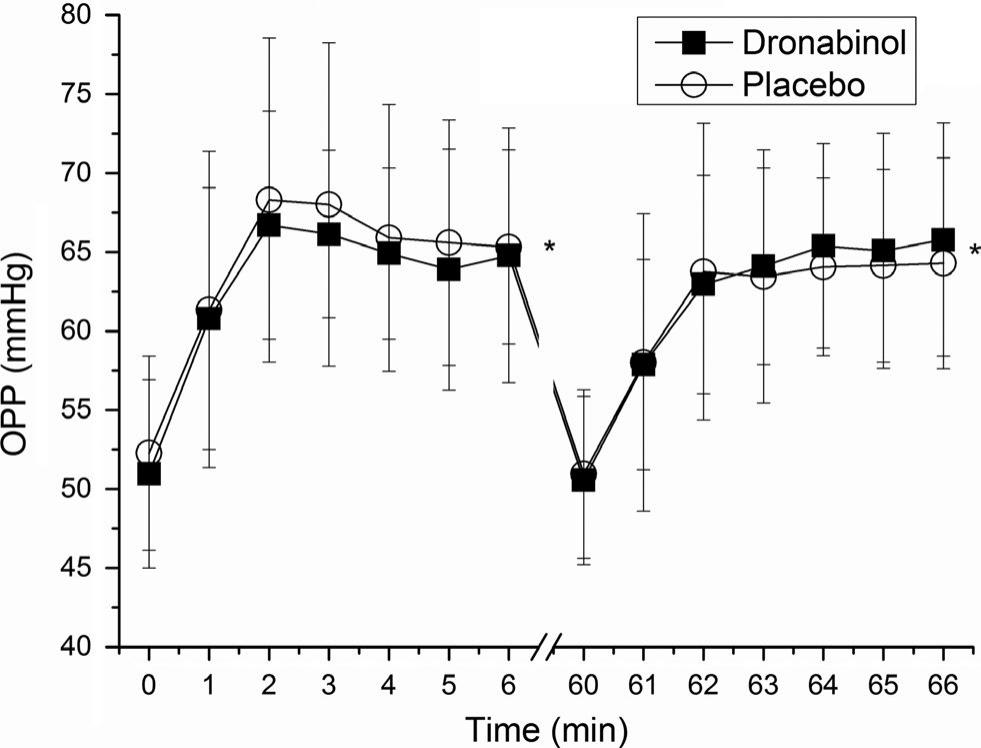
Change in ocular perfusion pressure (OPP) during isometric exercise. Minutes 0 and 60 represent values before start of isometric exercise. The first period of squatting was done without drug administration (minutes 1–6), and the second period after administration of placebo or dronabinol (minutes 61–66). Circles represent the placebo day whereas squares represent the dronabinol day. Data are presented as mean ± SD (*n* = 24). *Significant changes vs. baseline.

**Figure 4:**
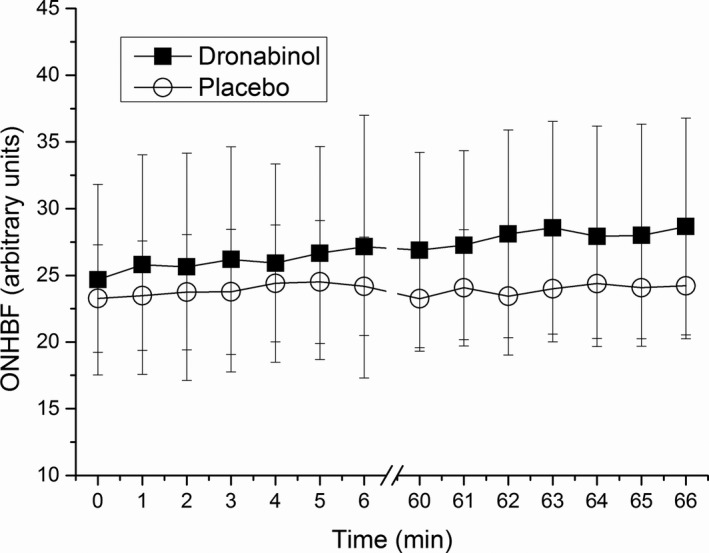
Change in optic nerve head blood flow (ONHBF) during isometric exercise. Minutes 0 and 60 represent values before start of isometric exercise. The first period of squatting was done without drug administration (minutes 1–6), and the second period after administration of placebo or dronabinol (minutes 61–66). Circles represent the placebo day whereas squares represent the dronabinol day. Data are presented as mean ± SD (*n* = 24).

**Figure 5:**
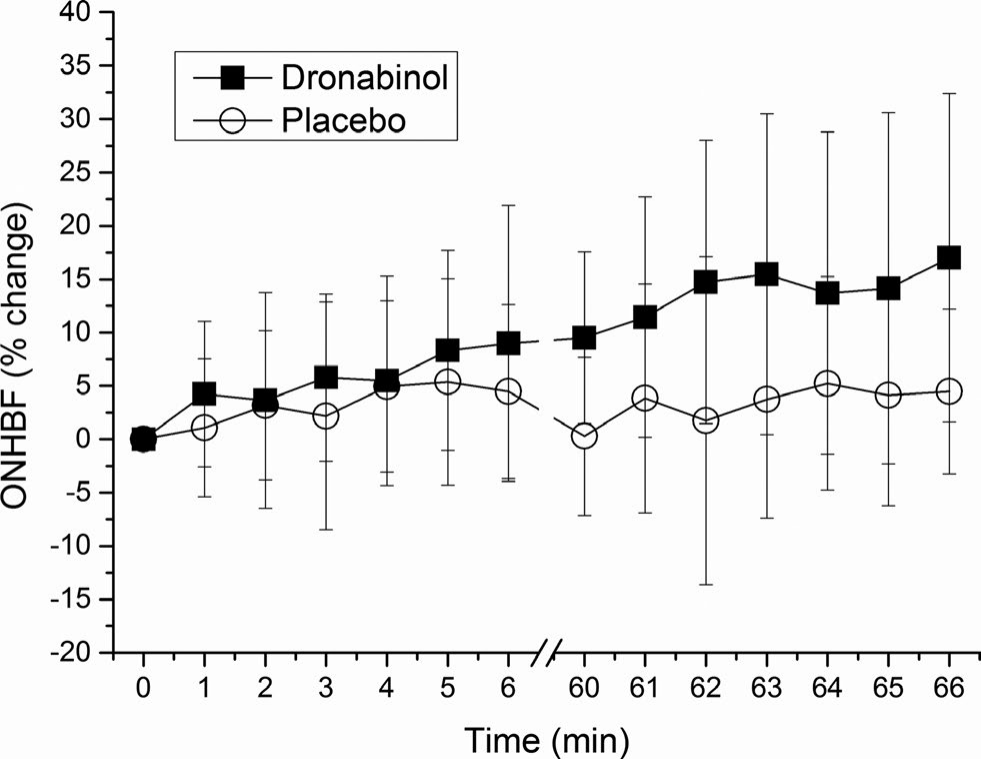
Relative change in optic nerve head blood flow (ONHBF) during isometric exercise when taking the resting predose values as baseline (minute 0). The first period of squatting was done without drug administration (minutes 1–6), and the second period after administration of placebo or dronabinol (minutes 61–66). Circles represent the placebo day whereas squares represent the dronabinol day. Data are presented as mean ± SD (*n* = 24).

During both pretreatment periods, isometric exercise induced a significant increase in MAP (*P* < 0.01 vs. baseline at each time point) and had no significant effect on IOP and, therefore, an increase in OPP was seen (*P* < 0.01 vs. baseline at each time point; **Figure** 3). The maximum increase in OPP during the pretreatment period was 31.4 ± 19.1% on the placebo day and 30.9 ± 13.4% on the dronabinol day (*P* = 0.67 between study days), respectively. This increase was paralleled by a maximum increase in ONHBF of 5.4 ± 9.7% and 9.0 ± 12.9%, respectively (*P* = 0.75 between study days, **Figures** 4 and 5). The increase in ONHBF was less pronounced than the increase in OPP indicating for some degree of autoregulation.

The maximum increase in OPP during isometric exercise was 26.1 ± 12.6% for the placebo and 30.1 ± 12.0% for the dronabinol day after drug administration. No significant difference in the time course of OPP in response to isometric exercise was seen after administration of either placebo or dronabinol (*P* = 0.67 between study days; **Figure** 3). The maximum increase in ONHBF was 4.6 ± 9.0 and 6.8 ± 11.5% after administration of placebo and dronabinol, respectively. This response of ONHBF to isometric exercise was also comparable between groups (*P* = 0.45 between study days; **Figures** 4 and 5).

**Figure 6:**
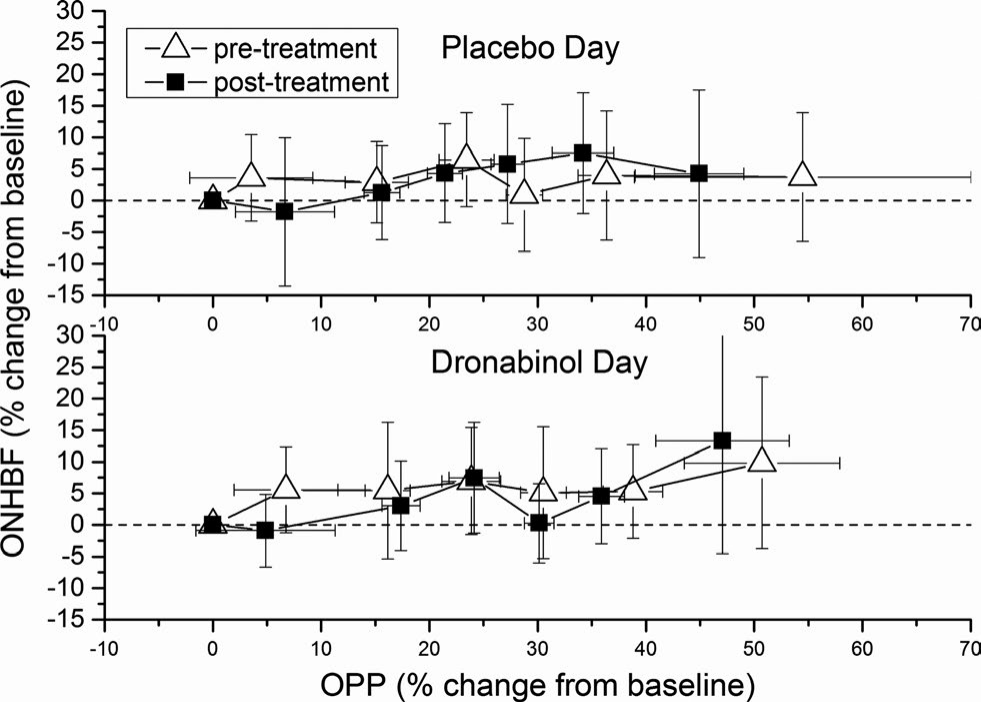
Pressure‐flow relationship determined by categorized ocular perfusion pressure (OPP) and optic nerve head blood flow (ONHBF) values during isometric exercise. Relative data were sorted into groups of 24 values, each according to ascending OPP. The dotted line indicates baseline values.

Pressure/flow relationships for both groups are presented in **Figure** 6. Neither placebo nor dronabinol had an impact on the pressure/flow relationship. ONHBF was regulated during the whole range of OPP observed in the present study, which was up to an increase of about 50% from baseline.

Interestingly, although ONHBF was significantly increased after administration of dronabinol, ONHBF regulation was not altered during isometric exercise, as illustrated in **Figure** 5.

## Discussion

Oral intake of 5 mg dronabinol was well tolerated and induced no psychoactive side effects in healthy volunteers. Although administration of dronabinol had no effect on systemic hemodynamics or IOP, it induced an increase in resting ONHBF by ~ 10%, which was significant as compared with placebo. No change was seen in the autoregulatory properties of the ONH vascular bed in response to an increase in OPP. In summary, to the best of our knowledge, this is the first study showing that low dose, orally administered dronabinol increases ocular blood flow without changing OPP.

It is known since the 1970s that cannabinoids can lead to a concentration‐dependent decrease in IOP up to 30% from baseline.^29^ Recently, the discovery of the endocannabinoid system in the eye has revived the interest in IOP‐independent functions of cannabinoid receptor agonists, in particular, in the cardiovascular effects of both synthetic and endogenous cannabinoids.^30^ Data from both *in vitro* as well as animal experiments support the hypothesis that cannabinoids act as vasodilators.^31^, ^32^ Further, data from isolated perfused retinal arterioles suggest that cannabinoids mediate changes in vascular resistance through endothelial receptor targets.^18^

Our data show that single administration of dronabinol leads to a significant increase in ONHBF in the order of 10% *in vivo*. In a previous self‐experiment, the cannabinoid delta‐9‐tetrahydrocannabinol was administered orally to eight healthy subjects, resulting in an increase of retinal blood velocity,^16^ but the study lacked a control group. The result of this small‐scale pilot study was now confirmed in a randomized, placebo‐controlled, double‐masked study design.

Interestingly, administration of dronabinol did not alter the pressure‐flow relationship during isometric exercise, although subjects started at higher baseline ONHBF levels (**Figure** 5). These experiments were done to test the hypothesis that the vasodilator dronabinol would counteract the vasoconstrictor response of the ONH vasculature to an increase in OPP. Our data do, however, confirm that during isometric exercise, the increase in ONHBF is less pronounced than the increase in OPP indicating for effective autoregulation. This is in agreement with previous results indicating the presence of blood flow autoregulation in the ONH.^4^, ^5^, ^6^, ^33^, ^34^ In patients with glaucoma, the response of ocular blood flow to isometric exercise has been consistently found to be altered during isometric exercise.^35^, ^36^ Whether dronabinol would normalize this abnormal autoregulatory response in glaucoma is still to be investigated.

Although our results indicate that orally administered dronabinol increases ONHBF, the molecular mechanism behind this effect warrants further investigation. As stated previously, it has been shown that activation of cannabinoid CB_1_ receptors leads to vasorelaxation and a decrease in vascular resistance.^37^, ^38^ This vasorelaxation seems to be mediated via the nitric oxide as well as the endothelin system, 38, 39 both of which are known to also play an important role in the regulation of vascular tone in the eye.^1^, ^3^, ^27^, ^28^, ^40^ To what extend the latter findings can be translated to the ocular vasculature needs to be further investigated. Data from isolated retinal arterioles show that the cannabinoid‐mediated vasorelaxation is abolished when the endothelium is removed, again indicating an important role of the nitric oxide/endothelin system in the vasoactive properties of cannabinoids.^18^

In contrast to previous studies where a significant reduction in IOP was found in both, patients with glaucoma and healthy subjects,^16^, ^41^, ^42^ no statistically significant change in IOP was observed in the present study. This can be well explained with the low dose of dronabinol used in the current study, which was considerably lower than in previous experiments and the fact that the subjects in our study started at lower IOP levels.^16^, ^41^, ^42^ The choice for the low dose of dronabinol in the current study was taken to investigate whether vasoactive properties can be observed without inducing changes in OPP.

Some limitations have to be considered when interpreting our results. First, as the study was conducted in healthy subjects, it remains unknown whether our conclusion also holds true in patients with vascular ocular disease or glaucoma. Thus, studies in patients are needed to see whether the blood flow effects are also present in patients with compromised blood flow and abnormal autoregulation. Further, the study used single administration of the drug and a low dose of the drug was chosen because of the reasons mentioned above. In addition, we cannot conclude of ocular hemodynamic effects of long‐term administration of dronabinol. Finally, it is not clear if or to what extent the observed changes in blood flow relate to a possible clinical effect. Longitudinal studies are warranted to investigate if the changes in perfusion are beneficial in terms of clinical outcome and/or glaucomatous progression.

The strength of our study is the randomized, double‐masked, placebo‐controlled design, which is currently considered as the gold standard for interventional trials. In particular, the two‐way crossover design, where every subject act as its own control, reduces the influence of confounding covariates and allows for detection of small effects even with limited sample sizes. Further, as for the current drug, a psychoactive activity cannot be fully excluded and may be anticipated by the subjects, the double‐masked design is of major importance. Double‐masked conditions were assured in the current study by manufacturing placebo capsules with the identical appearance as the active study drug.

In conclusion, the present data indicate that orally administered, low‐dose dronabinol increases ONHBF in healthy subjects without affecting IOP, OPP, or inducing psychoactive side effects. Further studies are needed to investigate whether this effect also occurs in patients with ocular vascular disease or glaucoma.

## Funding

This study was funded by the Austrian Science Fund (FWF) (KLI 340).

## Conflict of Interest

The authors declared no competing interests for this work.

## Author Contributions

N.H. and D.S. wrote the manuscript. N.H., G.G., L.S., and D.S. designed the research. N.H., M.K., S.S., S.P., K.S., M.B., M.A.‐T., and D.S. performed the research. N.H., R.M.W., L.S., G.G., and D.S. analyzed the data.
